# Benchmark of the Oxford Nanopore, EM-seq, and HumanMethylationEPIC BeadChip for the detection of the 5mC sites in cancer and normal samples

**DOI:** 10.3389/freae.2024.1362926

**Published:** 2024-07-25

**Authors:** Kseniia A. Deinichenko, Valentina G. Vynogradskaya, Pavel A. Grebnev, Valeriya M. Mikova, Dmitriy O. Bobylev, Abusaid M. Shaymardanov, Alexey A. Ivashechkin, Marina V. Erokhina, Alexandra I. Akinshina, Anna V. Semyanihina, Sergey I. Mitrofanov, Konstantin S. Grammatikati, Vladimir S. Yudin, Sergey M. Yudin, Antonida V. Makhotenko, Anton A. Keskinov, Sergey A. Kraevoy, Anna S. Makarova, Ekaterina A. Snigir, Dmitry V. Svetlichnyy, Veronika I. Skvortsova

**Affiliations:** ^1^ Federal State Budgetary Institution Centre for Strategic Planning and Management of Biomedical Health Risks of the Federal Medical Biological Agency (Centre for Strategic Planning of FMBA of Russia), Moscow, Russia; ^2^ Federal State Budgetary Institution N.N. Blokhin National Medical Research Cener of Oncology of the Ministry of Health of the Russian Federation (N.N. Blokhin NMRCO), Moscow, Russia; ^3^ Federal Medical Biological Agency (FMBA of Russia), Moscow, Russia

**Keywords:** DNA methylation, epigenetics, DNA modification, nanopore sequencing, DNA methylation detection, enzymatic methyl-sequencing

## Abstract

**Introduction:** Whole-genome DNA methylation identification is crucial for profiling physiologically and clinically relevant epigenetic changes. Although there are multiple experimental methods, their accuracy, advantages, and disadvantages need to be investigated in their application to complex tissue objects. In this study, we performed a benchmark of 5mC detection with Oxford Nanopore and enzymatic methyl-sequencing (EM-seq) methods.

**Material and Methods:** To this end, we profiled in a genome-wide manner 5mC sites in colorectal tumors and normal tissues for three patients and applied the HumanMethylationEPIC BeadChip as an additional control approach. We estimated the whole-genome scale of the methylation detection that each method yields.

**Results:** Our investigation describes the sensitivity and specificity of each platform and the impact that sequencing coverage brings. Our analysis revealed the higher sensitivity and specificity of Nanopore sequencing over the EM-seq method. Moreover, Oxford Nanopore Technology (ONT) sequencing, followed by Megalodon methylation detection, demonstrates better quantitative agreement of the epigenetic signals between biological replicates. Furthermore, our analysis highlights that with 40× and above coverage, EM-seq slightly outperforms ONT and yields highly accurate detection of the 5mC signals (AuPR = 0.99178 and AuROC = 0.98161).

**Conclusion:** The study was performed on colon cancer and adjacent normal tissue samples, placing our investigation close to the real application of methylation studies in oncology.

## 1 Introduction

Epigenetic modifications refer to changes in gene activity without any alterations in the DNA sequence, compared to genetic modifications such as nucleotide polymorphisms, insertions, and deletions. DNA methylation is one of the key epigenetic modifications, where a methyl group (-CH_3_) covalently attaches to the cytosine (C) at 5′-carbon, mostly in cytosines located in the CpG dinucleotides, also known as CpG sites. The human methylome consists of approximately 58 million CpG sites (Vaisvila et al., 2021) with a diverse regulatory function across the whole genome, which raises the important issue of the precise, high-throughput detection of epigenetic modifications to elucidate and interpret their role.

Usually, DNA methylation of the promoter region leads to expression repression (Greenberg and Bourc’his, 2019). However, there are examples where the methylation level and gene expression are positively correlated (Anastasiadi et al., 2018; Moarii et al., 2015), highlighting the complex regulatory aspects beyond a simple on/off model. There are several possibilities and proposed mechanisms that explain the effects brought associated with DNA 5mC modifications. In general, epigenetic changes in the DNA might shift protein–DNA interactions and modify the affinity of the loci to interact with nucleosome proteins, ultimately leading to expression changes.

The abnormal changes in methylation levels are among the common markers of the number of human cancers due to their impact on neoplastic transformation (González et al., 2021; Turpín-Sevilla, 2021; Nishiyama and Nakanishi, 2021). In order to elucidate the role of DNA methylation and its potential involvement in the processes leading to malignization, DNA methylation profiling has to be performed with high accuracy for both normal and tumor tissues. Modern methods to detect methylation rely on different experimental approaches and have their own limitations and detection possibilities. The most common method for 5mC detection is bisulfite sequencing (Ulahannan and Greally, 2015), which is able to detect 5mC at a single-base resolution along the whole genome. The limitation of the method is DNA susceptibility to damage during bisulfite conversion. Enzymatic methyl-sequencing (EM-seq) overcomes the issue using a set of two enzymatic reactions that protect the modified cytosines from deamination, resulting in non-methylated cytosine to uracil conversion and further detection of methylated cytosines (Vaisvila et al., 2021). EM-seq preserves the DNA backbone (Han et al., 2022), delivers higher library quality, outputs longer reads, and provides more uniform coverage even for the C-rich asymmetric unmethylated regions; this ultimately decreases false positive detection compared to bisulfite sequencing. Direct monomolecular sequencing based on the Oxford Nanopore Technology (ONT) approach became the state-of-the-art for the study of both large genomic alterations and chemical DNA modifications such as 5mC (Simpson et al., 2017; Wang et al., 2021). With the development of experimental and computational processing methods, the average sequence accuracy increased from 96.52% for R9.4.1 to 98.34% for R10.4 (Luo et al., 2022). However, the sequencing output for R10.4 is lower than that of the previous version (Ni et al., 2023), which is still intensively used in genome sequencing studies, especially considering the large amount of data already generated in genomics. Infinium MethylationEPIC Array 850K (EPIC) is another common method used for the high-throughput profiling of DNA methylation at predefined positions. It covers only 15% of the total methylome, which is mostly coding and enhancer regions (Moran et al., 2016). Furthermore, a microarray-based approach is applicable only to the human genome, but EM-seq and ONT can be applied to the DNA of any species.

In the current work, we compared three methods for DNA methylation detection applied to colorectal tumors and matched normal samples. We investigated genome-wide methylation detected with EM-seq and ONT. Furthermore, we performed profiling using HumanMethylationEPIC 850K microarrays as an independent platform, not relying on sequencing. We particularly selected both tumor and normal tissue samples for investigations because DNA methylation is one of the most studied modifications in oncogenomics with a high potential for translational research. In order to gain insight into the positive and negative sides of each approach, we also profiled biological replicate studies using tumor tissue from the same patient to obtain information about the variance in the methylation detection results across methods and propose an approach to investigate the sensitivity and specificity of the study techniques. In the current study, we focused on the main questions related to the performance of the EM-seq, ONT, and HumanMethylationEPIC chips with respect to methylation detection for cancer and normal tissue samples.

## 2 Materials and methods

### 2.1 Tissue sample collection

The sequencing samples, pairs of tumor tissues, and adjacent normal tissues were collected from the Federal State Budgetary Institution “NN Blokhin National Medical Research Center of Oncology” (NN Blokhin NMRCO) of the Ministry of Health of the Russian Federation. The resected samples were immediately stored at −80 ° . All the patients signed informed consent forms.

### 2.2 DNA extraction

Genomic DNA (gDNA) from the tissue samples was extracted using the QIAamp DNA Mini Kit (QIAGEN, Germany) in accordance with the manufacturer’s protocol. The yield and purity of the isolated gDNA were manually determined using the Quantus Fluorometer (Promega, United States) and NanoDrop OneC Microvolume UV-Vis Spectrophotometer (Thermo Fisher Scientific, United States), respectively. Only gDNA samples with absorbance ratios A260/280 of 1.7–1.9 and A230/260 of 1.8–2.2 were selected for further analysis.

### 2.3 Enzymatic methylation sequencing

Libraries for NGS-sequencing were constructed using the NEBNext^®^ Enzymatic Methyl-seq Kit (New England Biolabs, United States) in accordance with the manufacturer’s protocol. For the EM-seq experimental protocol, 100–200 ng of genomic DNA was subjected to ultrasonic fragmentation using the Covaris LE220-plus Focused-ultrasonicator (Covaris, United States), followed by library preparation of DNA shred to 270–320 bp on average. gDNA concentrations were measured using the Qubit 4.0 Fluorometer (Thermo Fisher Scientific, United States).

Samples were indexed using NEBNext Multiplex Oligos for Enzymatic Methyl-seq (unique dual index primer pairs), NEB #E7140. We performed DNA cytosine conversion control with unmethylated phage lambda (λ) DNA and CpG-methylated pUC19 DNA controls provided in the kit. Considering the obtained methylation values in controls compared to the recommended threshold, the conversion in the experimental samples was completed successfully. The size of the resulting libraries was determined using the Agilent D1000 Reagent Kit using Agilent 4200 TapeStation (Agilent Technologies, Inc., United States). The library pool was diluted to a final gDNA concentration of 1.5 nM prior to sequencing. Pool quality control was performed using the Agilent High Sensitivity D1000 ScreenTape Reagent Kit using Agilent 4200 TapeStation (Agilent Technologies, Inc., United States). Whole-genome sequencing was performed on Illumina NovaSeq 6000 (Illumina, Inc., United States) using the S2 Reagent Kit (Illumina, Inc., United States) upon 200 cycles with 2 × 100-bp paired-end reads.

### 2.4 Data processing for EM-seq

Raw sequencing reads were aligned to the reference genome (GRCh38) using Bismark v0.22.3 (Krueger and Andrews, 2011). Data for each sample were deduplicated, and cytosine methylation calling was performed on the deduplicated data. For each cytosine locus, the β -value was calculated as the ratio of the number of methylated reads to the total number of reads for the CpG locus.

### 2.5 Human Methylation EPIC 850K

Bisulfite conversion was performed using the DNA Zymo Methylation Kit (Zymo Research, United States) for 1,000 ng of genomic DNA. The following preprocessing steps were automated using the Tecan Freedom EVO using the Illumina Infinium MethylationEPIC BeadChip Kit (Illumina, United States) according to the manufacturer’s instructions (Infinium HD Methylation Assay Protocol Guide [15019519 v07]). Microarrays were scanned using the Illumina iScan System with the AutoLoader 2.× loading module.

### 2.6 Data processing for EPIC

The Infinium MethylationEPIC Array (Illumina, United States) was used to analyze the methylation status of 866,554 CpG sites, according to the manifest file (Infinium MethylationEPIC v1.0 B5). Data were processed using the minfi package (Fortin and Triche, 2017). The samples were quantile normalized, and probes with a signal detection *p*-value above 0.01 were removed from the analysis. For the remaining 835,521 CpGs, β -value and M-value were calculated. We used the sva package for batch effect analysis and data correction on the M-values (Leek et al., 2012).

### 2.7 Nanopore sequencing

For Nanopore sequencing, 1,500 ng of genomic DNA was taken for each sample. The Ligation Sequencing Kit SQK-LSK109 (Oxford Nanopore Technologies, United Kingdom) was used to prepare libraries. Sample preparation was carried out according to the manufacturer’s protocol (Genomic DNA by Ligation, version GDE_9063_v109_revY14Aug 2019). The libraries were sequenced on a PromethION 48 (Oxford Nanopore Technologies, United Kingdom), and the loading concentration per well was 50 pM.

### 2.8 Data processing for Nanopore

The obtained sequencing results, FAST5 files for each of the samples, were processed using the Megalodon (version 2.3.4) tool (Megalodon 2.3.3 Documentation, 2023) using the default parameters according to the manual with GRCh38 as the reference genome. As Megalodon performs simultaneous basecalling of raw reads, as well as methylation calling, it requires a basecalling model with read filtration based on quality (reads with Q-score >7 were considered for further analysis). There is a specialized model that takes into account the cytosine methylation provided by Rerio $\left(--guppy-configres\text{\_}dna_{r}941\text{\_}prom\text{\_}modbases\text{\_}5mC\text{\_}CpG\text{\_}v001.cfg\right)$ . The Megalodon result files consist of a basecalled FASTQ file, mapping bam files, and bedMethyl file with information about the 5mC positions (location and methylation levels). Apart from Megalodon, methylation calling from Nanopore data was performed using the Nanopolish tool (Simpson et al., 2017; Simpson et al., 2017), and the −−call−methylation subprogram using FASTQ files was generated by guppy_basecaller (version 5.0.11) (de.NBI Nanopore, 2023).

### 2.9 Methylation analysis and visualization

For each sample, four methylation files were generated with methylated position results from EM-seq, Nanopore (Megalodon and Nanopolish), and EPIC each. The methylation profiles were further compared in RStudio using the GenomicRanges (Lawrence et al., 2013), ChIPSeeker (Yu et al., 2015), and UpSetR packages (Conway et al., 2017). The plots for the CpG site intersection were plotted using the R density function. The performance of the whole-genome methods in terms of specificity and sensitivity was evaluated using the area under the receiver operating characteristic curve (ROC AUC) and precision-recall (PR) as implemented in the scikit-learn Python library (Pedregosa et al., 2011). For Gene Ontology (GO) analysis, we applied the enrichGO function as implemented in the clusterProfiler package (Wu et al., 2021). For the correlation, we used the cor function from the stats R package (R-core, 2013). Visualization was performed using the corrplot R package (Wei et al., 2022). Differential methylation analysis was performed using the methylKit R package (Akalin et al., 2012).

### 2.10 Genotyping of the cancer samples

We utilized the ability of Nanopore sequencing to catch genetic variants, particularly single-nucleotide polymorphisms (SNPs). This procedure was performed with Clair3 using GRCh38 as the reference (Zheng et al., 2022). The results of variant calling were used to search for associations with specific colorectal cancer syndromes. The obtained variants were annotated using the Ensembl Variant Effect Predictor (McLaren et al., 2016); then, the analysis of the detected variants for genotype–phenotype associations in the group of genes associated with the development and progression of colorectal cancer was performed by a medical geneticist. The described procedure was performed on two sample pairs, for which the whole dataset (ONT, EM-seq, and EPIC 850K) was obtained.

## 3 Results

### 3.1 Distribution of the 5mC sites detected with ONT, EM-seq, and EPIC in the hereditary colorectal tumor and normal tissue genomes

Here, we profiled both tumor and adjacent normal tissue samples of colorectal cancer (Figure 1A). We applied variant calling for ONT and identified that samples contain variants known for hereditary colon cancers (Supplementary Table S1). In the study, we aimed to compare whole-genome methylation detection methods. In order to estimate advantages and limitations, we benchmarked the methods based on a quantitative comparison of the number of CpG sites detected by each of the approaches. Considering the previous computational comparison of the Nanopore methylation calling methods (Liu et al., 2021), we performed 5mC calling from FAST5 files using Megalodon (Megalodon 2.3.3 Documentation, 2023) and Nanopolish (Simpson et al., 2017).

**FIGURE 1:**
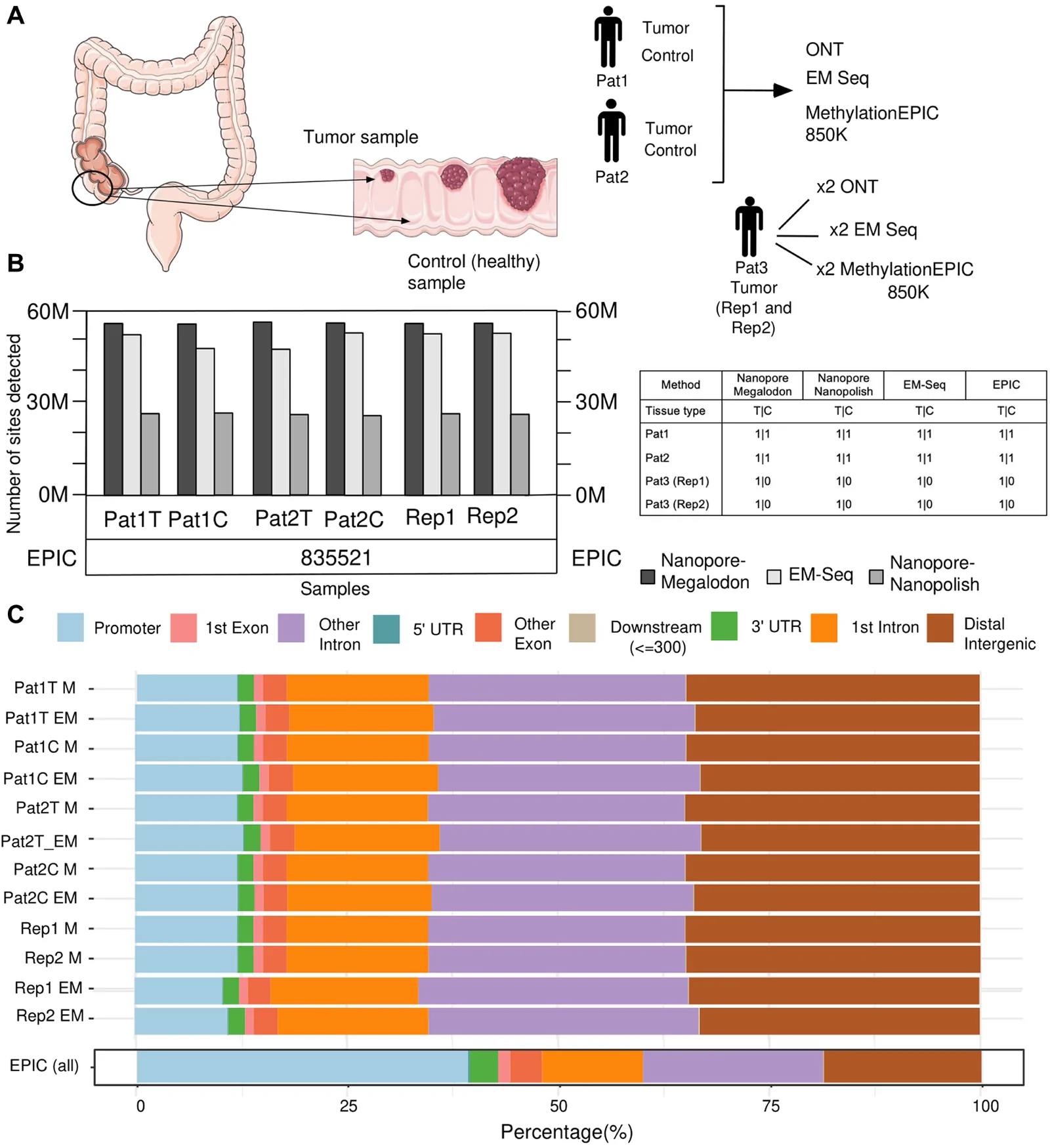
Overview of the research design and statistics on the number of detected methylated positions and their distribution in the genomic regions. **(A)** Summary of the experimental design. **(B)** Bar plot visualization for a number of 5mC sites detected from each sample by whole-genome methods (Nanopore + Megalodon, Nanopore + Nanopolish, and EM-seq). Number of sites detected by EPIC shown under the bar plot due to the array design feature. **(C)** Bar plot visualization of the distribution of detected 5mC sites by genomic regions.

We obtained the number of detected ( β -value >0.0) 5mC sites (Figure 1B). Both whole-genome methods detected a comparable number of methylated CpG sites, ranging from 48 to 54 million. Furthermore, the detected methylated CpG positions yielded minor quantitative differences between tumor and normal pairs. The methylation calling method for Nanopore sequencing was selected based on previously reported performance (Liu et al., 2021), where Megalodon was identified as the most optimal approach. Moreover, for all generated Nanopore data, our results also confirm that Nanopolish identifies significantly fewer sites, demonstrating the lower sensitivity of the approach than that of EM-seq in a genome-wide manner (Figure 1B).

The EPIC capacity of CpG detection yields more than 830 K (Figure 1B) positions for every microarray experiment. This amount is very close and strictly limited by the manufactured design of the platform (McEwen et al., 2018), which can identify up to 866,554 positions and is located mostly in promoter regions (Figure 1C). Furthermore, for whole-genome sequencing methods, both ONT–Megalodon and EM-seq yield similar distributions of the detected methylated CpGs, along with the annotation of the genomic elements (Figure 1C). Furthermore, we analyzed how each method performs within known genomic segments (promoters, introns, and distal intergenic regions) and identified that ONT + Megalodon yields higher-scoring ( β -value >0.9) 5mC sites across all types of loci (Supplementary Figure S3). Our analysis identified that both ONT + Megalodon and EM-seq demonstrate the highest performance with respect to the number of detected methylated positions. Nanopolish captured much fewer 5mC CpG sites than EM-seq and Megalodon. However, the distribution of the detected positions in the genomic regions qualitatively is very similar between all whole-genome approaches, with minor differences between normal and corresponding tumor samples.

### 3.2 5mC sites detected across samples and methods

Next, we compared the CpG sites detected in tumor and normal samples. To this end, we investigated matched tumor and normal pairs where methylation has been measured with all three experimental methods (Figure 2A).

**FIGURE 2:**
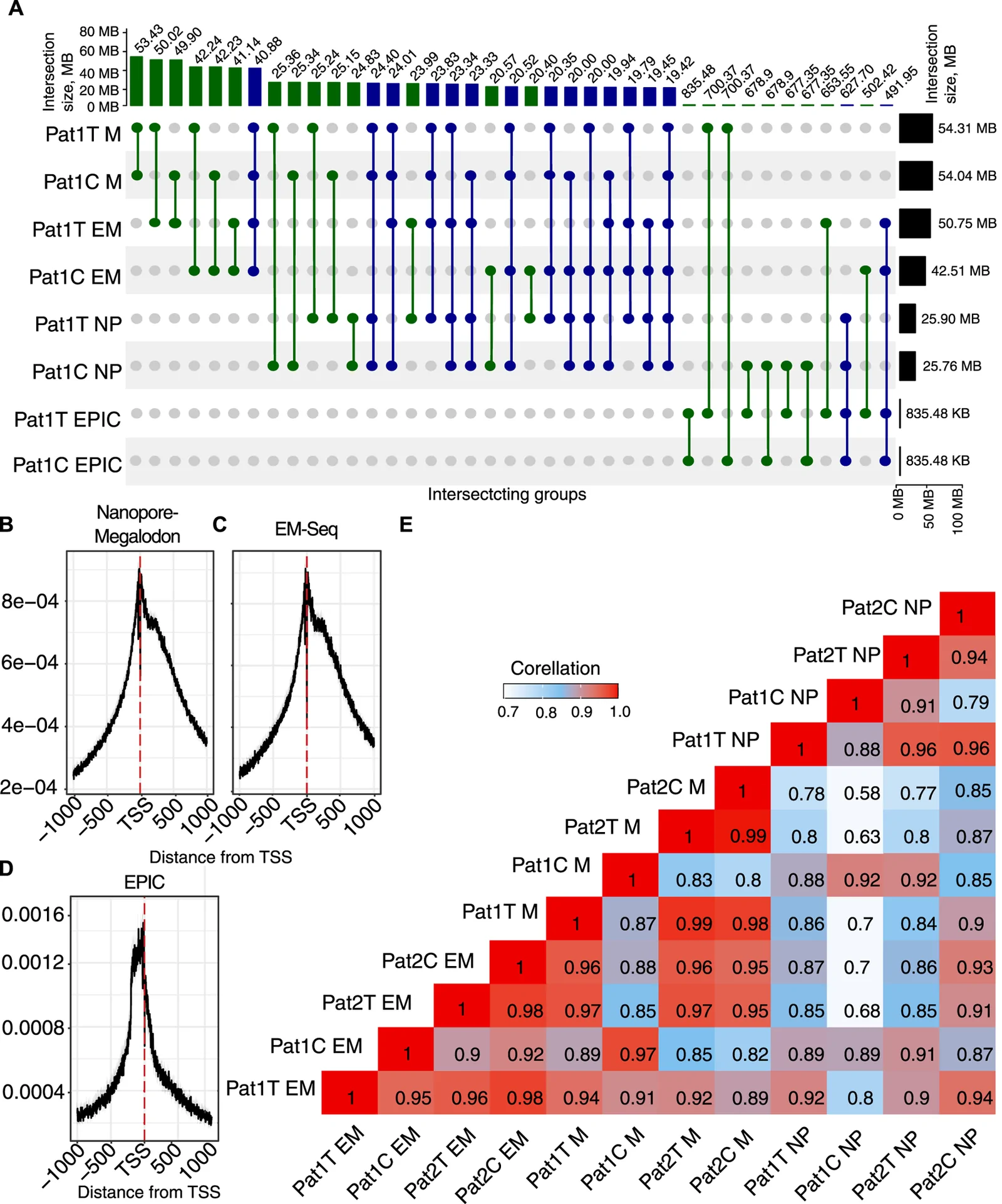
Overview of the methylation profile for the paired tumor/normal samples. **(A)** UpSet diagram showing intersection sets between methylation detection methods (M, Megalodon; EM-seq; NP, Nanopolish, and EPIC) in tumor (Pat1 T) and adjacent normal tissues (Pat1 C). **(B–D)** Density of 5mC ( β -value >0.0) around the transcription start site. **(E)** Pearson’s correlation plot of the methylation scores across methods and samples.

Our results indicate very high agreement on the total number of CpG sites detected with ONT + Megalodon and EM-seq. ONT + Megalodon identified 53.4 M common methylated sites between tumor and normal tissue, and EM-seq detected 41.14 M. Here, we hypothesize the lower sensitivity of the EM-seq method because we consider all CpGs with β -value >0.0, and for the same tumor, ONT + Megalodon and EM-seq yield nearly 50 M. Furthermore, an overlap between EPIC and ONT + Megalodon (700 K for tumor tissue) outperforms EPIC and EM-seq (635 K for tumor tissue). However, when the Nanopolish method was used, a significant decrease in sensitivity was observed compared to both EM-seq and Megalodon, which also agrees with previous benchmarking results (Liu et al., 2021). For the matched tumor/normal pairs of samples, Nanopolish detected only 24.83 M common methylated regions. Nanopolish and EM-seq together detected a lower number of methylated positions (20 M) compared to the common sites between Megalodon and EM-seq (50 M).

To investigate differences brought by the application of Megalodon or Nanopolish for the number of detected methylated sites, we applied EPIC results for the same biosamples. Surprisingly, when we compared Nanopore-based methods with EPIC, we identified that Megalodon yields a higher number of common methylated CpG sites (700 K). However, for the same tumor replicate, Nanopolish and EPIC have only 677 K in common. This indicates a much higher sensitivity of the ONT + Megalodon combination and allows us to conclude about the lower performance of the Nanopolish approach.

In addition to considering the common potentially methylated sites across computational and experimental methods, we also considered other properties of the methylation signal important for biological interpretation and usage of the methods. When we compared the distribution of the methylated ( β -value >0.0) CpG sites around the transcription start site (TSS), both ONT (Figure 2B) and EM-seq (Figure 2C) showed high similarity for profiles. However, the distribution of the methylation signal for EPIC (Figure 2D) follows the location of the designed probes for microarray.

In order to assess quantitative agreement across methods, we also performed a correlation analysis (Figure 2E). The result shows an expected grouping of the most correlated pairs in sub-clusters based on the processing method. This indicates obvious biases brought by every methylation detection algorithm. However, across all methods, the correlation remains very high. Our results indicate that EM-seq has lower sensitivity than ONT.

### 3.3 Biological replicate analysis yields the identification of the method with the lowest variance

Next, we investigated the general reproducibility of the results both in terms of the total number of identified methylated CpG sites and quantitative signal agreement across experiments. Two tumor samples obtained from one individual underwent methylation detection by the same pool of methods (EM-seq, ONT–Megalodon, ONT–Nanopolish, and HumanMethylationEPIC 850K). We identified very high agreement on the number of detected methylated CpG sites ( β -value >0.0) across biological replicates (Figure 3A). Moreover, we found that ONT + Megalodon is the most sensitive approach, with the largest number of detected sites. Furthermore, more than 98% of the 5mC-modified positions were common for two replicates processed with ONT + Megalodon. EM-seq is the second-most sensitive detection method in our study that successfully identified more than 51 M methylated positions with 97% common sites across biological replicates. However, ONT + Nanopolish detected almost two-fold fewer methylated sites than Megalodon, and 95% were consistent between replicates. Nanopolish yielded a much lower agreement on the 5mC detection in the replicate analysis given the same sequencing data. The results obtained with EPIC show very high agreement between replicates (almost 100%) and indicate a much higher overlap across replicates for 5mC sites detected with Megalodon rather than Nanopolish.

**FIGURE 3:**
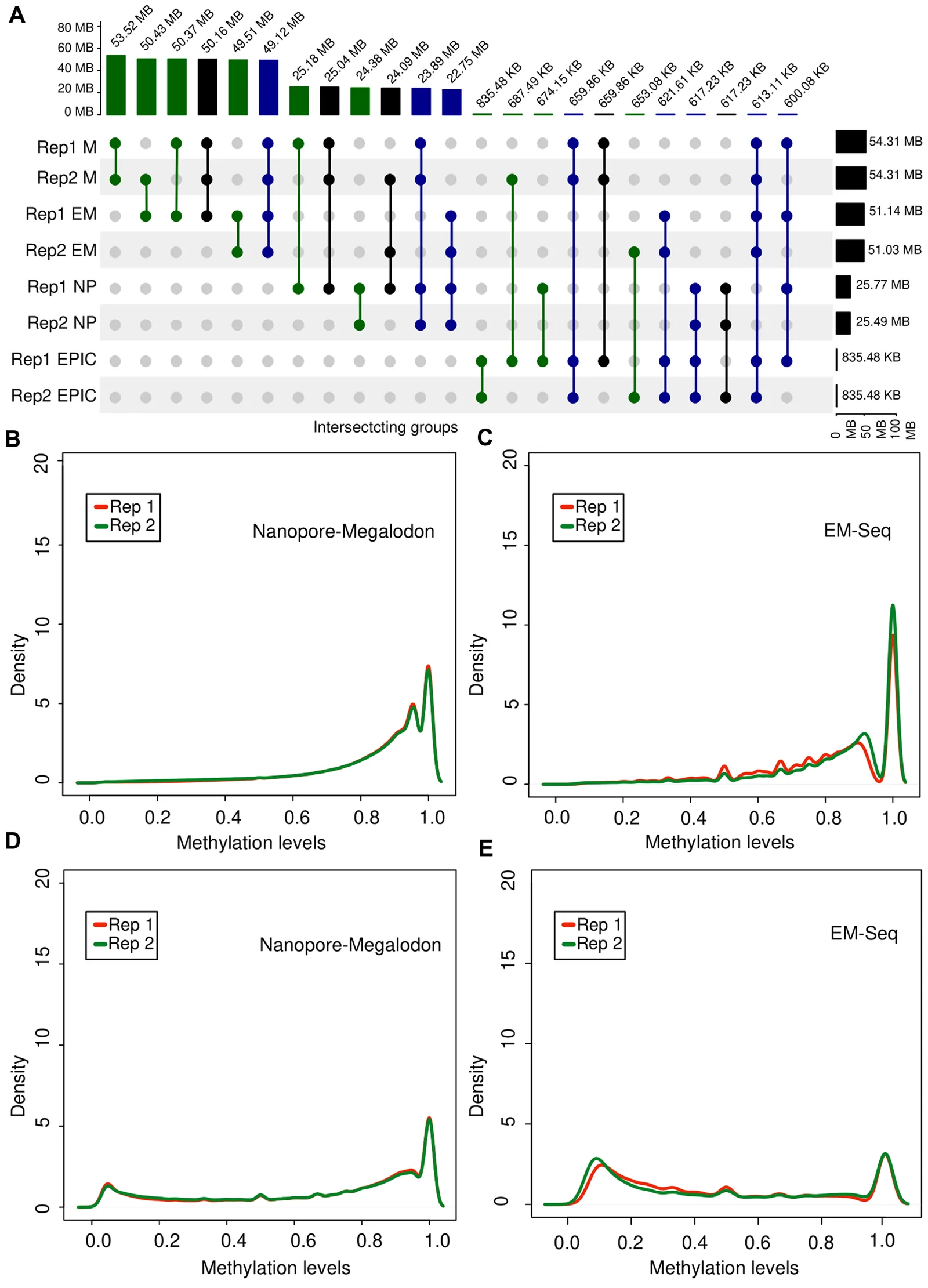
Overview of methylation profile consistency in biological replicates of tumor tissue. **(A)** UpSet diagram generated from the intersection sets between methylation detection methods for two biological replicates (Rep1 and Rep2) of tumor tissue. **(B,C)** Distribution of β -values across methods for biological replicates. Density plot of the β -values for the CpG sites common across all replicates and methods. A subset of methylation sites detected in each of the replicates by both whole-genome methods, ONT + Megalodon and EM-seq, was generated. β -values obtained from ONT + Megalodon in selected sites were plotted. **(C)** Density plot of the β -values (EM-seq) in sites common to all replicates and methods. Same as **B**, distribution of β -values obtained from EM-seq in the common selected methylation sites was plotted. The qualitative behavior of the curves demonstrates a higher variance of the results obtained with EM-seq than those with ONT. **(D)** Distribution of β -values in sites detected in both replicates by either Nanopore or EM-seq. Distribution of β -values in sites detected across both replicates by ONT + Megalodon but not captured by EM-seq. **(E)** Distribution of β -values in sites detected in both replicates by EM-seq but not captured by ONT + Megalodon. β -values in sites common in both ONT + Megalodon. Only sites common in both EM-seq and not detected by any ONT + Megalodon were taken into account.

Despite the high agreement of the tools with respect to the detection of the same 5mC positions in biological replicates, we investigated how scores of the methylation signal are distributed for each sample depending on the experimental and computational approach to obtain an insight into the variance brought by each method. To this end, we selected two sets of positions. We selected a set of methylated sites common for both biological replicates detected with both ONT + Megalodon and EM-seq. Next, we qualitatively investigated the density plot of the beta-values of each group. We identified that the ONT + Megalodon (Figure 3B) results for a set of common methylated CpG sites have a higher similarity of the detected epigenetic profiles than EM-seq results (Figure 3C), where density curves on the plot were less overlapping, indicating higher variance of the method itself.

In addition to investigations of the recurrence across replicate positions for ONT and EM-seq, we also examined the CpG sites that were found methylated in both replicates only with one of the approaches but were not detected by another method. Density curves obtained for ONT + Megalodon scores are more overlapping than those obtained for EM-seq, again indicating a higher variance of the EM-seq method (Figures 3D, E).

### 3.4 Sensitivity and specificity of the study methods

Our analysis identified the higher sensitivity of the ONT + Megalodon approach to capture more 5mC sites than EM-seq (Table 1). In order to gain insight into the overall sensitivity and specificity of the whole-genome methylation capturing possibilities, we again applied an approach based on the analysis of biological replicates. To this end, we selected a collection of the true positive (TP) (captured with EPIC in both replicates) and true negative (TN) positions (not methylated in both biological replicates as found with EPIC). CpG positions from EPIC with a methylation β -value lower than 0.3 were determined to be unmethylated. TP outcomes were defined as detected CpG sites found in both biological tumor replicates with β -values of more than 0.3 (600,000), while all the others were allocated as TN outcomes (250,000). Next, we used EM-seq and ONT (both with Nanopolish and Megalodon) to calculate the area under the ROC (Figure 4A) and PR curve (Figure 4B) using a β -value threshold as a variable parameter. We identified that ONT + Megalodon had the highest detection abilities both for sensitivity and specificity (AuROC = 0.97724 and AuPR = 0.99031). Moreover, ONT + Nanopolish demonstrated the lowest performance (AuROC = 0.94142 and AuPR = 0.95228) and was outperformed by EM-seq (AuROC = 0.96521 and AuPR = 0.98393).

**TABLE 1:** Sequencing coverage in biological replicates. The average coverage for the whole genome and CpG sites were calculated.

	ONT Rep1	EM-seq Rep1	ONT Rep2	EM-seq Rep2
Median total	49.30	21.60	49.80	21.40
Median CpG	23.10	18.20	23.80	22.00

**FIGURE 4:**
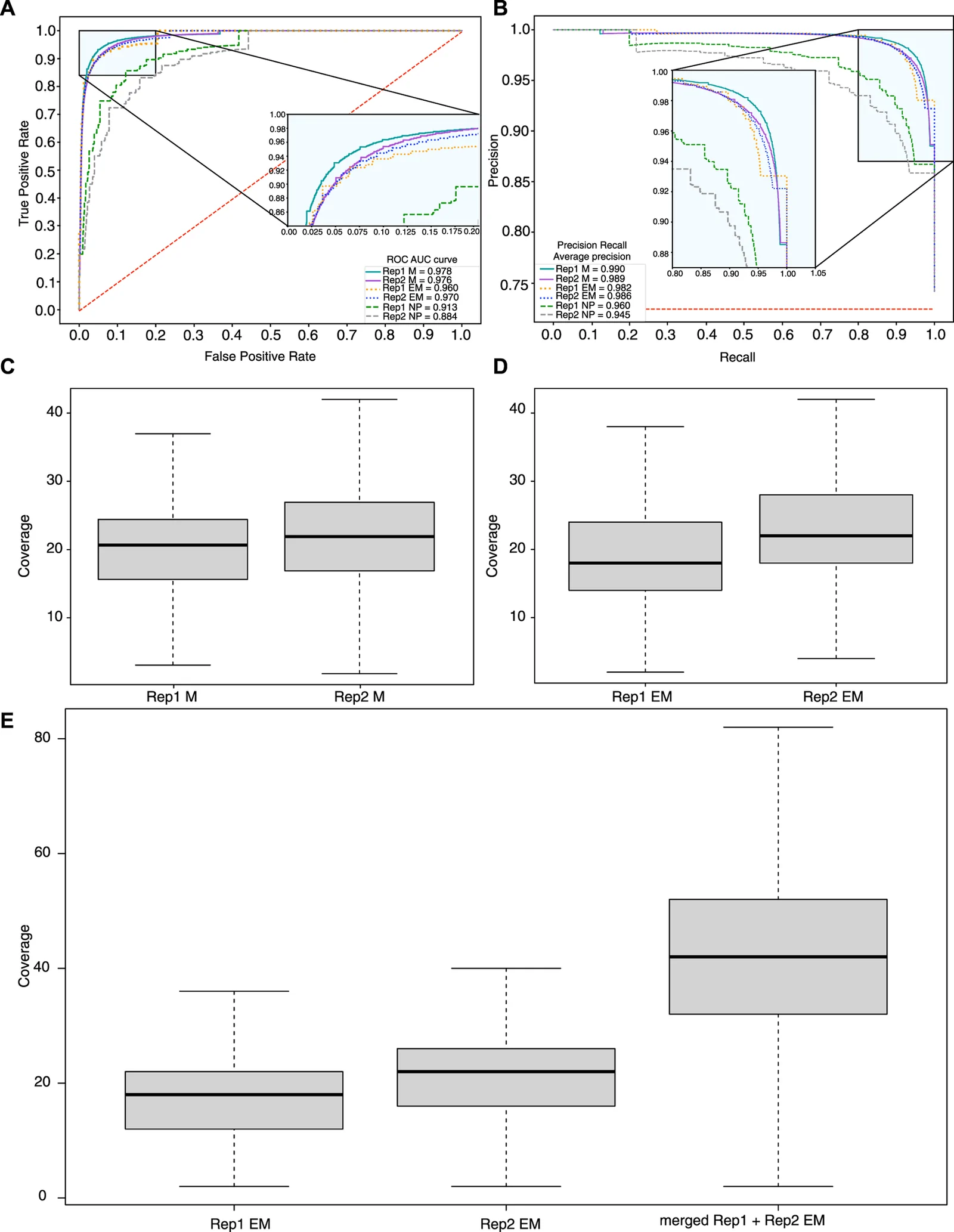
Sensitivity and specificity of methylation calling methods. **(A)** ROC and precision recall **(B)** curves calculated based on the methylation profiles obtained with ONT + Megalodon, ONT + Nanopolish, and EM-seq. A set of true positives and negatives was obtained based on the EPIC 850K array. As true positives, we used methylated ( β -value >0.3) in both replicates, and negatives are positions not methylated in both replicates as obtained with EPIC 850K. **(C)** Box plots for coverage of the CpG ( β -value >0.3) positions obtained with ONT and **(D)** EM-seq. **(E)** Box plot for coverage obtained for sites detected by whole-genome methods ( β -value >0.3) and not detected by EPIC considering only overlapping sites between platforms.

One of the main factors that can contribute to the sensitivity and specificity of methylation detection is sequencing coverage. Our results indicate that the median coverage of the EM-seq experiments for the study samples is 18–22×, and for ONT (filtered reads with quality >7), it is 23× (Figures 4C, D) for CpG sites. One of the advantages of EM-seq is its ability to detect CpG methylation even with coverage as low as 5× (Vaisvila et al., 2021). Furthermore, the ONT sequencing results indicate that 20× coverage is sufficient to capture methylation signals with high accuracy (Table 1).

In order to take into account sequencing depth and its impact on the comparison of the results obtained between EM-seq and ONT, we performed several computational experiments. We evaluated how an increase in coverage affects the detection of methylation with EM-seq. To this end, we merged the sequencing results of the two tumor biological replicate samples and performed methylation calling. Despite the relatively low coverage, approximately 50 million sites were detected. We investigated how the coverage affects the methylation detection ability. The coverage of individual samples was approximately 20× for each replicate, so after merging, the total coverage became nearly 40× (Figure 4E) for CpG sites and reached average ONT coverage (40×). We performed methylation calling on a merged sample and identified that the number of detected methylated CpG sites increased (from 51 M to 52 M) with higher coverage. For individual samples of EM-seq, 1,032,160 CpG sites were not detected in either of the 2 replicates but were detected in both Nanopore replicates. After merging 2 individual EM-seq samples, 871 sites were detected out of 1,032,160. Thus, the merging of samples led to a two-fold increase in coverage, but in terms of CpG sites, detection does not yield a big difference.

We used ROC and PR metrics to estimate both the sensitivity and specificity of the merged EM-seq data with high CpG coverage. The results showed significant improvement of the EM-seq results (AuPR = 0.99178 and AuROC = 0.982) by slightly outperforming the ONT + Megalodon results (AuPR = 0.990 and AuROC = 0.977) (Supplementary Figures S5, S6). Our results indicate that ONT + Megalodon show the best performance in terms of the detection of true methylation sites given similar coverage of the CpG positions with EM-seq.

### 3.5 Differential DNA methylation

DNA methylation studies usually rely on statistical approaches to identify a highly confident subset of the CpG sites with differential methylation between the study conditions. We performed differential methylation calling using methylKit (Akalin et al., 2012). Using EM-seq, we identified 1,463 CpG positions of differential methylation between tumor and normal samples. ONT + Megalodon found 1,061 CpG sites with significant changes in the 5mC level. Unfortunately, there is no overlap between the positions obtained for the two experimental approaches. One of the most obvious explanations is low statistical power due to an insufficient number of samples. Next, we assigned sites in promoters to genes, and for ONT + Megalodon, we found 307 hypermethylated and 506 hypomethylated genes. EM-seq detected 46 hypermethylated genes and 29 hypomethylated genes in the tumor. We performed GO enrichment analysis, and the results did not include specific cancer-related terms that indicate the high false positive/false negative detection rate (Supplementary Table S4), given the low number of samples.

To investigate the agreement of the approaches to capture differentially methylated positions, we performed a custom analysis. We studied how EM-seq and ONT methods agree on the sites with high changes in the methylation signal. To this end, we used samples from two patients where both normal and adjacent tissues have been profiled with two genome-wide approaches. We identified a set of positions where the detected delta of methylation signals between normal and cancer tissues was maximal (the absolute difference of β -values is equal to ± 1.0). Surprisingly, for this analysis, we identified only minor agreement (Figure 5A, B) between EM-seq and ONT (672 and 681 sites for each patient). These findings again highlight the importance of using replicates in order to avoid biases due to high variance. The results obtained with EM-seq demonstrate a much higher number of differentially methylated positions and confirm the higher variance of the EM-seq approach that identified fold times more positions with highly changed methylation levels than ONT but only with few in common.

**FIGURE 5:**
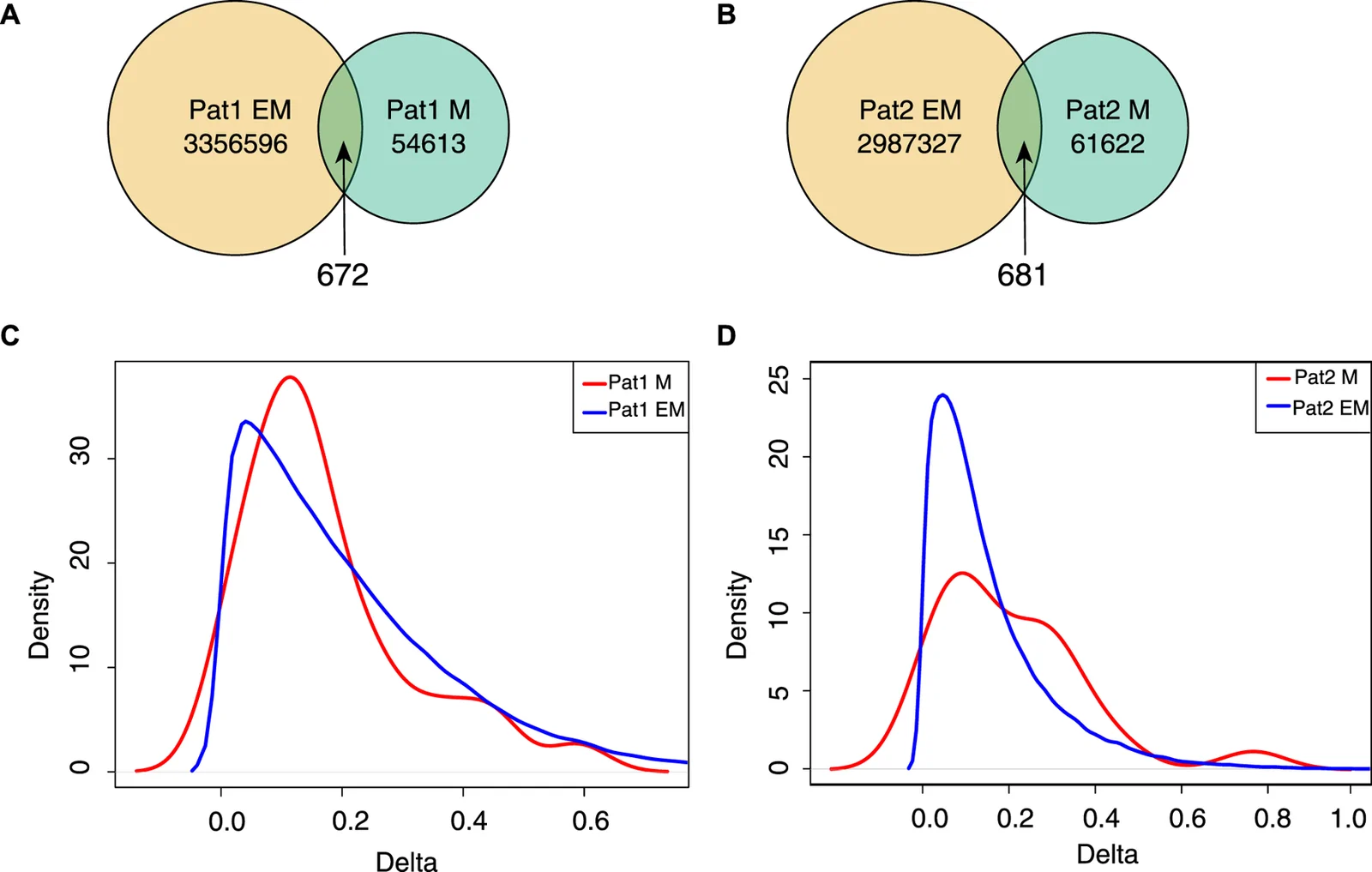
Sites with high (+-1.0) delta values of the methylation signal between tumor and normal samples. **(A,B)** Venn plot for strongly changed methylation sites detected for each patient with ONT + Megalodon and EM-seq. **(C,D)** Density plot of the deltas for β -values based on the EPIC results for differentially methylated positions as obtained with the whole-genome methods (ONT + Megalodon or EM-seq).

We applied EPIC to quantify methylation differences for highly changed sites detected with ONT or EM-seq (Figures 5C, D). The density profile indicates that the majority of highly changed sites do not show high methylation changes in EPIC (Figures 5C, D). Taking into account the high discordance of the results, we conclude that the usage of replicates is the right way to identify sites with methylation changes, and relying on single-sample profiling is prone to yielding false positive/negative results.

Overall, our analysis indicates that both ONT and EM-seq have high variance, and identification of the differential methylation sites requires more replicates (biological or technical). Furthermore, the ONT + Megalodon approach is less prone to have overestimated differences in methylation signals. Moreover, analysis of promoter methylation did not yield enrichment of the GO terms associated with cancer processes, which indicates the requirement to apply more data samples to gain high-confident results.

## 4 Discussion

DNA methylation (5mC) is one of the main epigenetic modifications that ultimately have regulatory effects on gene expression and cell phenotype (Greenberg and Bourc’his, 2019; Nishiyama and Nakanishi, 2021). A large variety of experimental methods for 5mC detection raises important issues about the consistency of the results, which experimental approach to use considering biosample type/quality, and research questions. Whole-genome bisulfite sequencing (WGBS) and its improved modifications, such as EM-seq, are a very popular and widely used technique both for fundamental and medicine-oriented research. EM-seq outperforms WGBS as it requires a low amount of input DNA, yields higher library quality, and provides more uniform coverage (Han et al., 2022). Direct DNA sequencing using ONT, coupled with computational data processing, yields a map of the epigenetic modifications along the genome. ONT requires a large amount of input DNA because it is PCR-free and based on direct sequencing. HumanMethylationEPIC 850K Array represents a high-quality methylation detection method and is considered the gold standard for DNA methylation studies.

We designed an experimental setup to evaluate EM-seq, ONT, and HumanMethylationEPIC 850K Array in order to highlight the strong and weak points of each approach. To assess the performance of the methods, we profiled both normal and adjacent tumor tissues of patients with hereditary colon cancer (Supplementary Table S1). Moreover, one tumor sample was profiled twice with the three methods to obtain well-verified estimations of the methylation signal variability across biological replicates. Our experimental design relies on tissue samples, in contrast to other similar studies where cell lines have been used (Foox et al., 2021).

We analyzed genome-wide methylation profiles for tumors and adjacent healthy tissues using Nanopore and EM-seq. In general, ONT + Megalodon, EM-seq, and 850K EPIC yield highly accurate and reproducible results. Nanopore sequencing showed better performance in terms of sensitivity, specificity, and consistency than HumanMethylation EPIC chips. We investigated the coverage impact on methylation detection. Our results demonstrated the expected improvement in both the sensitivity and specificity for recovering true 5mC modification signals when using consistent sites across biological replicates obtained using the 850K EPIC platform. We identified that a median coverage of 15–17× for EM-seq is suitable to accurately capture (AuROC = 0.96521 and AuPR = 0.98393) true methylation profiles. Furthermore, a coverage increase of up to 40× yielded improvement, reaching AuROC = 0.98161 and AuPR = 0.99178. Such coverage twice overcomes the output based on the standard protocol. We hypothesize that ONT would also increase the performance given higher sequencing coverage. Furthermore, given the similar coverage of the CpG sites, ONT outperforms EM-seq. Here, we focused on the recommended coverage, which is supposed to be obtained by spending a unit of the reagents per sample according to prescribed protocols.

We investigated the performance of the ONT using two processing algorithms—Megalodon and Nanopolish. Overall, in line with previous studies (McLaren et al., 2016), we confirm that Megalodon outperforms other methods. The lower performance of the Nanopolish can be explained based on the way it scores methylation signals. Nanopolish uses a hidden Markov model that relies on locus information about CpG sites and detected signals. To call 5mC sites, Nanopolish yields a log-likelihood ratio of 5mC versus an unmethylated state (Simpson et al., 2017). To this end, Nanopolish groups close CpG sites and detect methylation jointly so that each site within a group obtains the same methylation status. Megalodon performs methylation calling for each CpG independently, relying on a neural network model that leads to improvements over scoring with the hidden Markov model.

Computational processing of the ONT sequencing results has disadvantages due to the generation of a large amount of raw data, followed by highly intensive computations for processing and methylation detection. From an experimental point of view, ONT relies on direct DNA sequencing, which restricts the use of small biosamples. However, while EM-seq is a method for 5mC and 5hmC detection, ONT can be used to retrieve other DNA modifications apart from 5mC, as well as genetic variants such as single-nucleotide variants or structural variants. Overall, we highlighted the strengths and limitations of ONT and EM-seq methods (Table 2), investigated the accuracy of 5mC detection depending on the coverage, tested various methylation calling models, and performed benchmarks relying on the microarray platform with proven detection accuracy.

**TABLE 2:** Summary of the comparison of characteristics and performance of DNA methylation detection methods based on literature search and obtained benchmarking results.

Characteristic	EM-seq	Nanopore	EPIC
Whole-genome method	Yes	Yes	Yes
Accuracy	High	High	High
Dispersion	Average	Low	Low
Amount of data	Average	Large	Small
Ability to detect other modifications	Only 5hmC	Yes	No
Computational expenses	Average	High	Low
Ability to detect genomic variations	No	Yes	No

Altogether, combining the experimental results obtained with the three benchmarked experimental approaches for two tumor biological replicates, we estimated the sensitivity and specificity for whole-genome methylation methods and identified the ONT + Megalodon approach as the best performing in terms of sensitivity and specificity.

Our work was performed on the newly generated data for tissue samples, which is preferred over cell lines for the estimation of method limitations under biologically complex conditions with high variability. This raises additional difficulties and adds additional noise to the data. However, we performed the analysis for the tissue biomaterial because it better reflects the possible medical applications of the methods. We expect more applications of ONT sequencing in biological and medical studies to uncover complex relationships between mutation patterns and epigenetic profiles.

## Data Availability

The original contributions presented in the study are publicly available. This data can be found here: https://cloud.cspmz.ru/s/JBJegJcRgX9MqBt.
