# Supplementary material for: Benchmark of the Oxford Nanopore, EM-seq, and HumanMethylationEPIC BeadChip for the detection of the 5mC sites in cancer and normal samples
Source: Front Epigenet Epigenom. 2024 Jul 25;2:1362926. doi: 10.3389/freae.2024.1362926 (PMC13639291; doi:10.3389/freae.2024.1362926)
Supplement: Supplementary file 1 [file DataSheet2.PDF]

Benchmark of the Oxford Nanopore, EM-seq, and HumanMethylationEPIC BeadChip for detection of the 5mC sites in cancer and normal samples

1 SUPPLEMENTARY DATA

Table S1. Genetic variants potentially associated with colorectal cancer development detected in analyzed samples.

Patient	Associated syndromes	Identified variants
Pat1	C18.7 Localized sigmoid cancer, stage I (cT1N0M0), colorectal oligopolyposis	VUS POLE (Rs201115064 III class; Ht)
Pat2	C18.4 Metastatic adenocarcinoma of the transverse colon with invasion into the stomach, stage IVA (T4bN1bM1a) (HEP: S6)	mutMLH1 (rs587778885 V class; Ht)

Table S2. QC for EM lambda and phage and pUC19 methylation.

Sample	Number of sequenced reads	Number of aligned reads	CpG methylation in Lambda	CpG methylation in pUC19
Pat1T	405382887	307406536	0.26%	97.16%
Pat1C	214944585	175451281	0.5%	97.4%
Pat2T	202285257	163151077	0.4%	98.2%
Pat2C	386966285	325366915	0.1%	94.84%
Rep1	365339583	284152791	0.11%	94.27%
Rep2	479251731	322225963	0.07%	97.0%

Table S3. Average coverage for whole genome.

Sample	Coverage (ONT)	Coverage (EM-Seq)
Pat1T	46.60	10.58
Pat1C	44.90	8.84
Pat2T	61.70	8.25
Pat2C	45.30	9.66
Rep1	49.30	10.80
Rep2	49.80	10.70

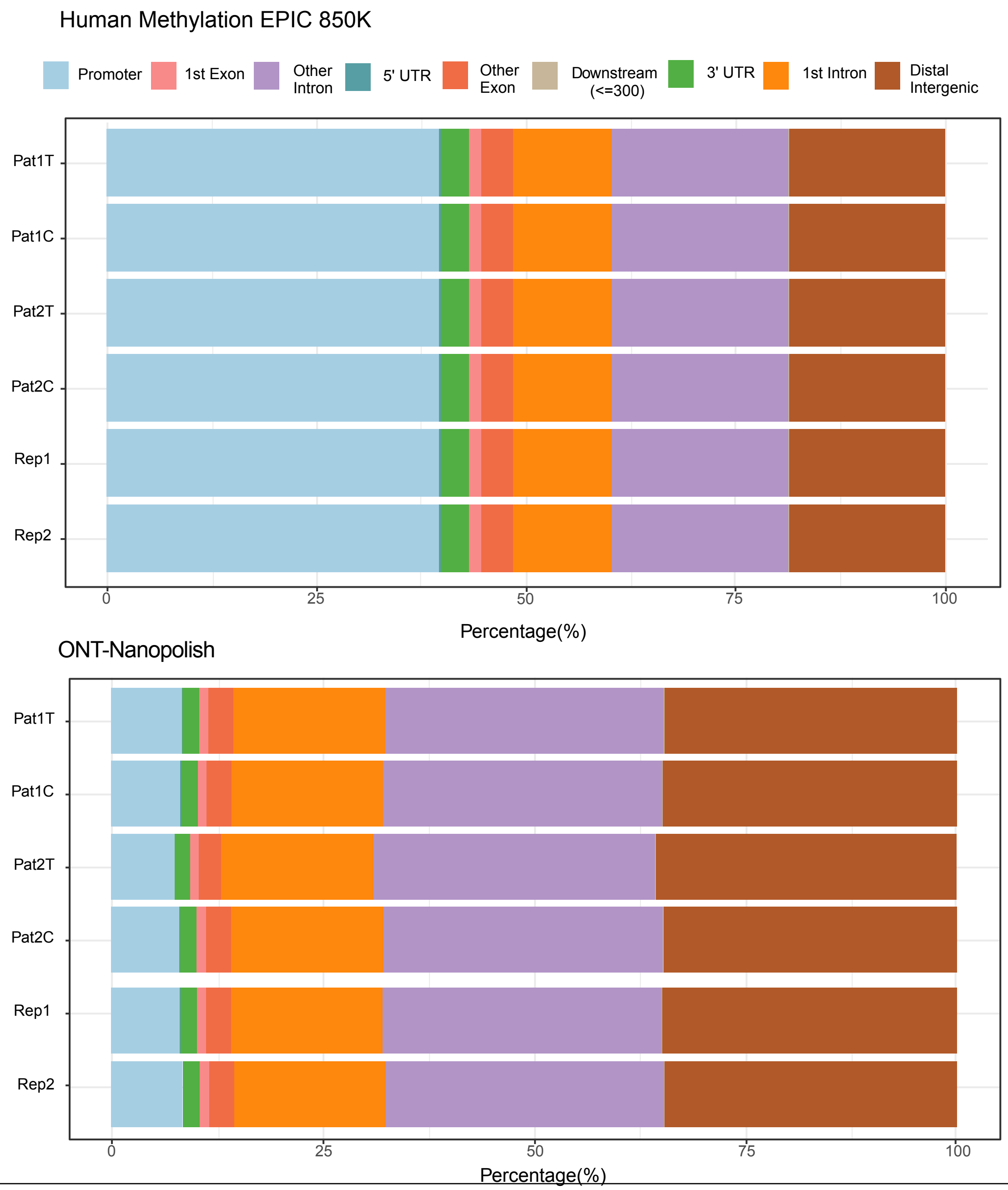

Figure S1. Genomic region annotation for individual ONT+Nanopolish samples and Human EPIC

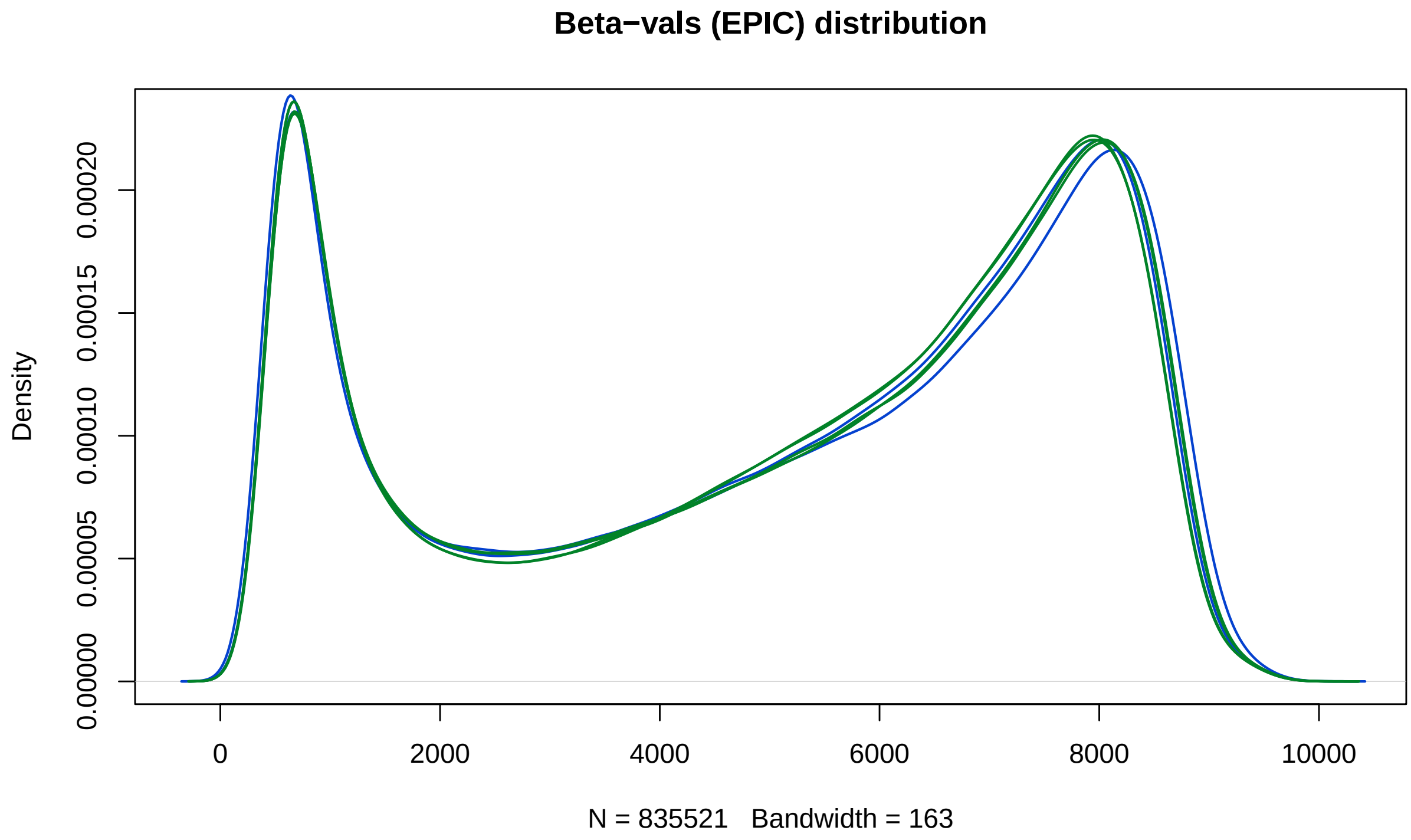

Figure S2. Methylation levels (β-value) distribution in processed Human Methylation EPIC samples

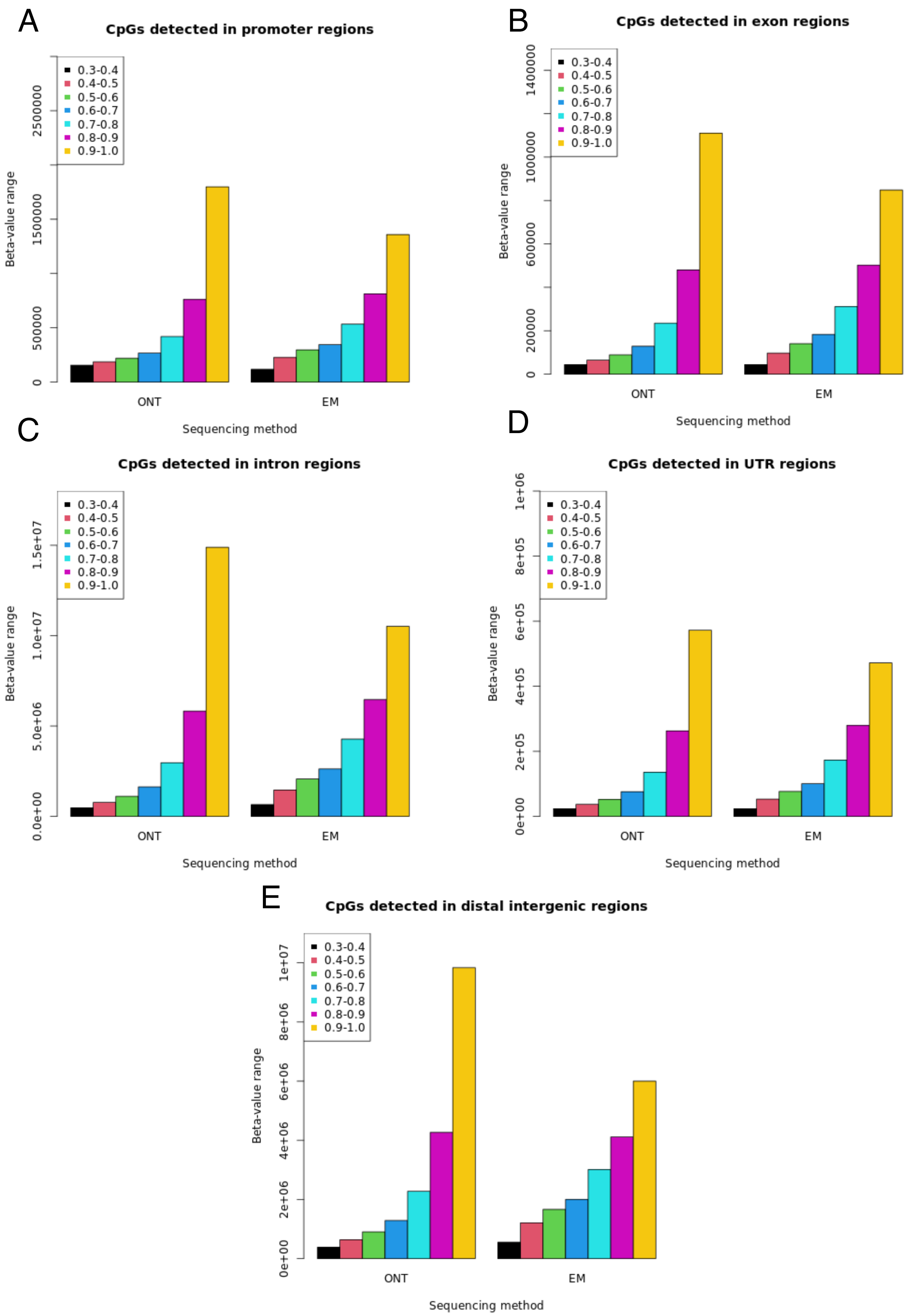

Figure S3. Methylation levels (β-value) distribution across genomic regions annotated by ChIPseeker detected by ONT(Megalodon) and EM-seq in tumor tissue (Rep1-2)

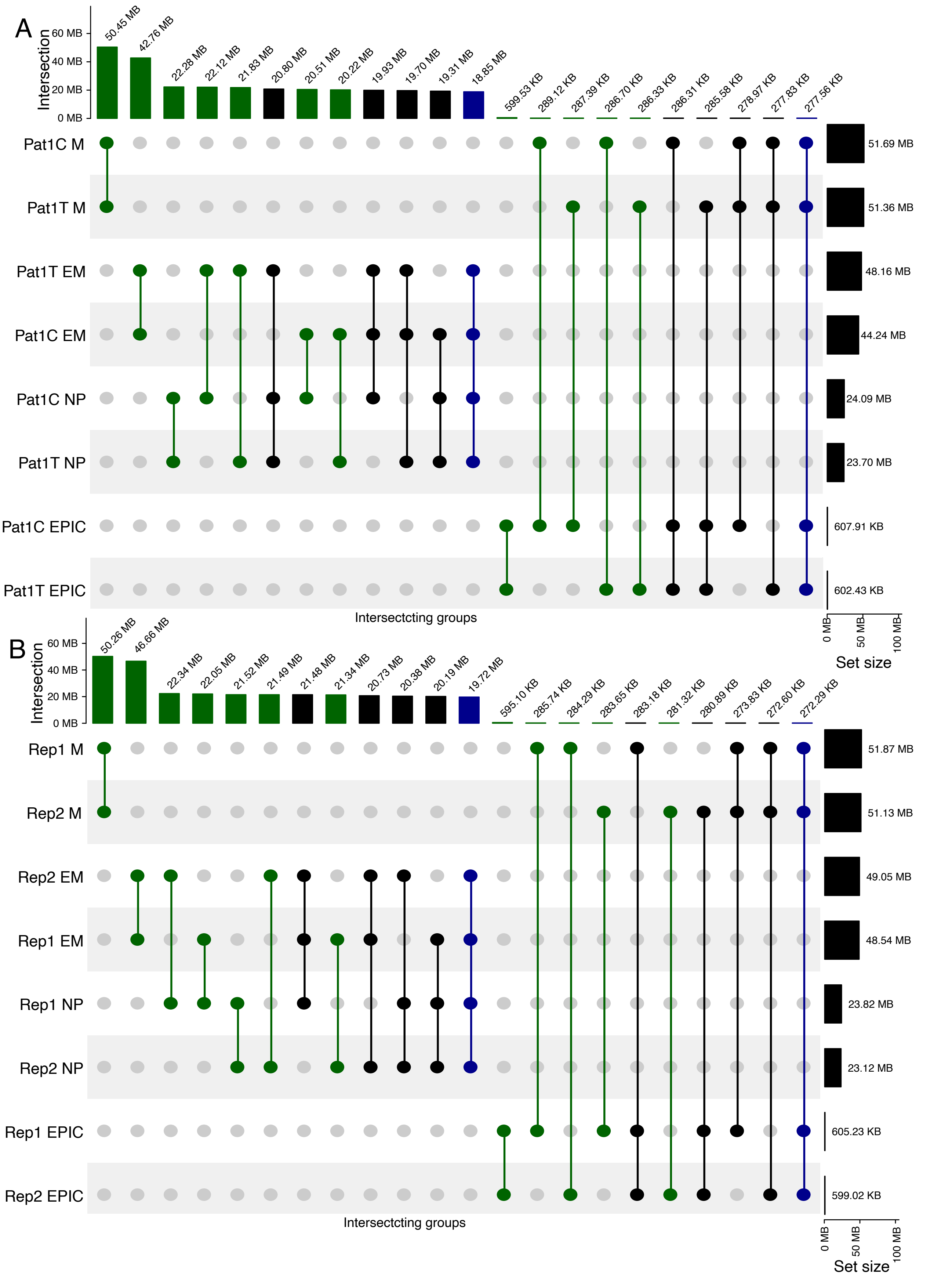

Figure S4. UpSet diagram showing intersection sets between methylation detection methods (M - Megalodon, EM-Seq, NP - Nanopolish, EPIC) in tumor (Pat1 T) and adjacent normal tissue (Pat1 C) with methylation level detected (β-value >0.3)

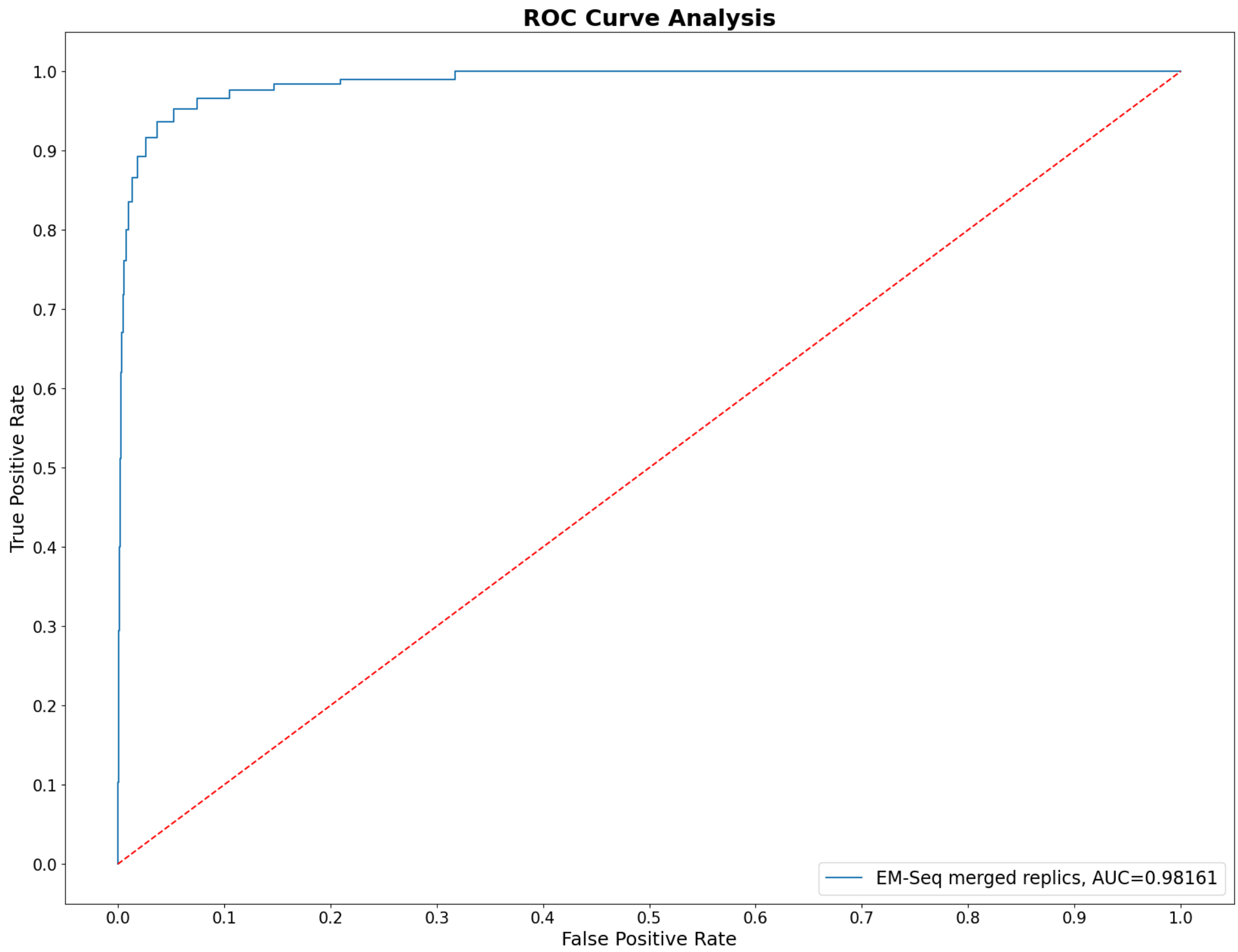

Figure S5. ROC curve for merged EM-Seq replica

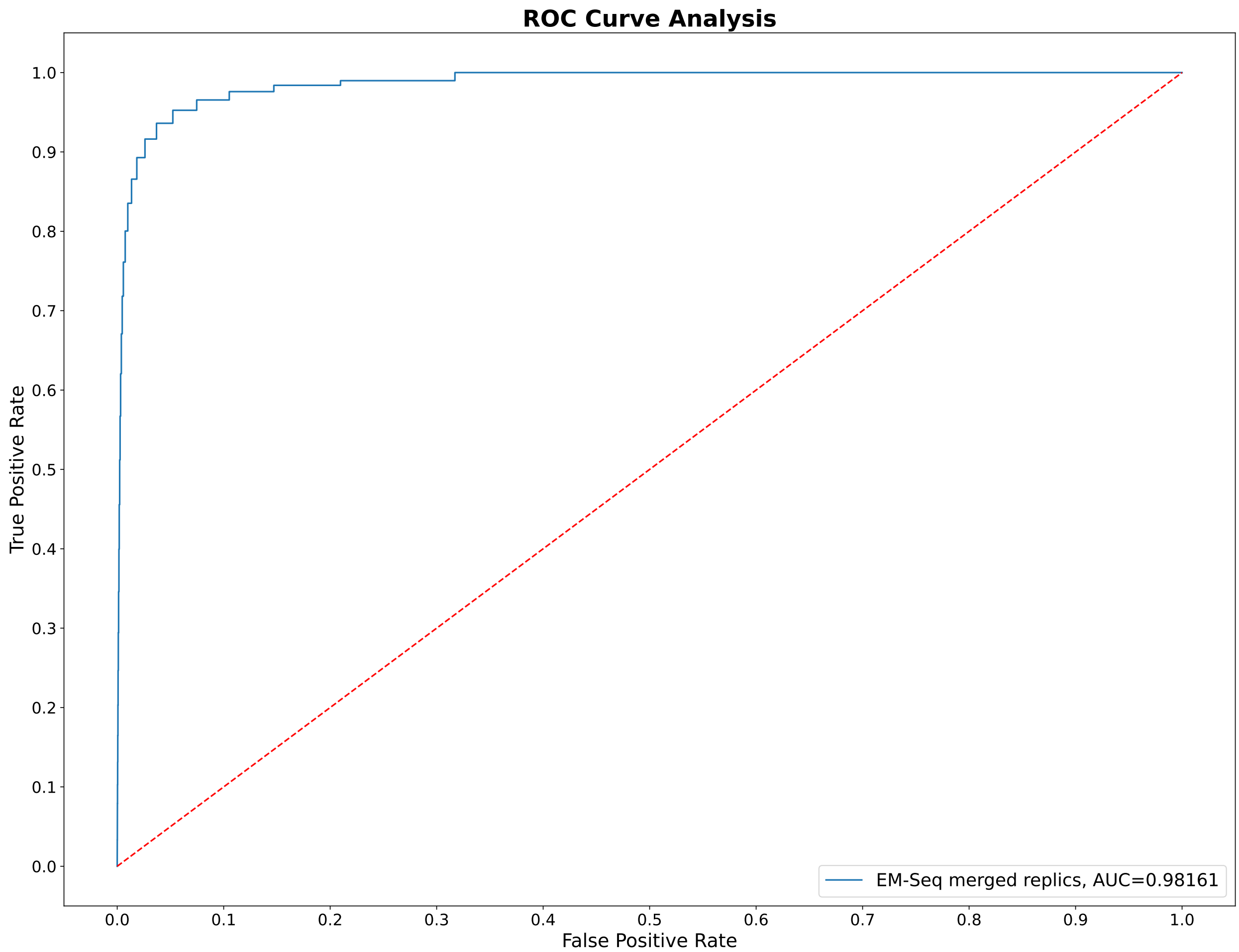

Figure S6. PR curve for merged EM-Seq replica

Table S4. GO terms for differentially methylated genes annotated to sites detected by Methylkit. Hypermethylated genes in ONT+Megalodon.

GO term	Ontology	Description	qvalue
GO:0098742	BP	cell-cell adhesion via plasma-membrane adhesion molecules	0.0002
GO:0007156	BP	homophilic cell adhesion via plasma membrane adhesion molecules	0.0009
GO:0001708	BP	cell fate specification	0.0325

Table S5. GO terms for differentially methylated genes annotated to sites detected by Methylkit. Hypomethylated genes in ONT+Megalodon.

GO term	Ontology	Description	qvalue
GO:0098742	BP	cell-cell adhesion via plasma-membrane adhesion molecules	0.0049
GO:0007409	BP	axonogenesis	0.0052
GO:0007157	BP	heterophilic cell-cell adhesion via plasma membrane cell adhesion molecules	0.009
GO:0007411	BP	axon guidance	0.009
GO:0007157	BP	neuron projection guidance	0.009
GO:0061564	BP	axon development	0.009
GO:0003156	BP	regulation of animal organ formation	0.0326
GO:0140058	BP	neuron projection arborization	0.0338

Table S6. GO terms for differentially methylated genes annotated to sites detected by Methylkit. Hypermethylated genes in EM-seq.

GO term	Ontology	Description	qvalue
GO:0006488	BP	dolichol-linked oligosaccharide biosynthetic process	0.0045
GO:0006490	BP	oligosaccharide-lipid intermediate biosynthetic process	0.0045
GO:0046527	MF	glucosyltransferase activity	0.0009

Table S7. GO terms for differentially methylated genes annotated to sites detected by Methylkit. Hypomethylated genes in EM-seq.

GO term	Ontology	Description	qvalue
GO:0004843	MF	cysteine-type deubiquitinase activity	0.0112
GO:0101005	MF	deubiquitinase activity	0.0110
GO:0004197	MF	cysteine-type endopeptidase activity	0.0013
GO:0019783	MF	ubiquitin-like protein peptidase activity	0.0117
GO:0008234	MF	cysteine-type peptidase activity	0.0182
GO:0005001	MF	transmembrane receptor protein tyrosine phosphatase activity	0.0249
GO:0019198	MF	transmembrane receptor protein phosphatase activity	0.025
